# Sticking With It: ER-PM Membrane Contact Sites as a Coordinating Nexus for Regulating Lipids and Proteins at the Cell Cortex

**DOI:** 10.3389/fcell.2020.00675

**Published:** 2020-07-22

**Authors:** Mohammad F. Zaman, Aleksa Nenadic, Ana Radojičić, Abel Rosado, Christopher T. Beh

**Affiliations:** ^1^Department of Molecular Biology and Biochemistry, Simon Fraser University, Burnaby, BC, Canada; ^2^Department of Botany, University of British Columbia, Vancouver, BC, Canada; ^3^The Centre for Cell Biology, Development, and Disease, Simon Fraser University, Burnaby, BC, Canada

**Keywords:** endoplasmic reticulum, plasma membrane, ER-PM contact sites, membrane tethers, extended synaptotagmins, VAP (VAMP-associated protein), membrane stress, phospholipid regulation

## Abstract

Membrane contact sites between the cortical endoplasmic reticulum (ER) and the plasma membrane (PM) provide a direct conduit for small molecule transfer and signaling between the two largest membranes of the cell. Contact is established through ER integral membrane proteins that physically tether the two membranes together, though the general mechanism is remarkably non-specific given the diversity of different tethering proteins. Primary tethers including VAMP-associated proteins (VAPs), Anoctamin/TMEM16/Ist2p homologs, and extended synaptotagmins (E-Syts), are largely conserved in most eukaryotes and are both necessary and sufficient for establishing ER-PM association. In addition, other species-specific ER-PM tether proteins impart unique functional attributes to both membranes at the cell cortex. This review distils recent functional and structural findings about conserved and species-specific tethers that form ER-PM contact sites, with an emphasis on their roles in the coordinate regulation of lipid metabolism, cellular structure, and responses to membrane stress.

## Introduction: ER-PM Membrane Contact Sites (MCSs)

**FIGURE 1:**
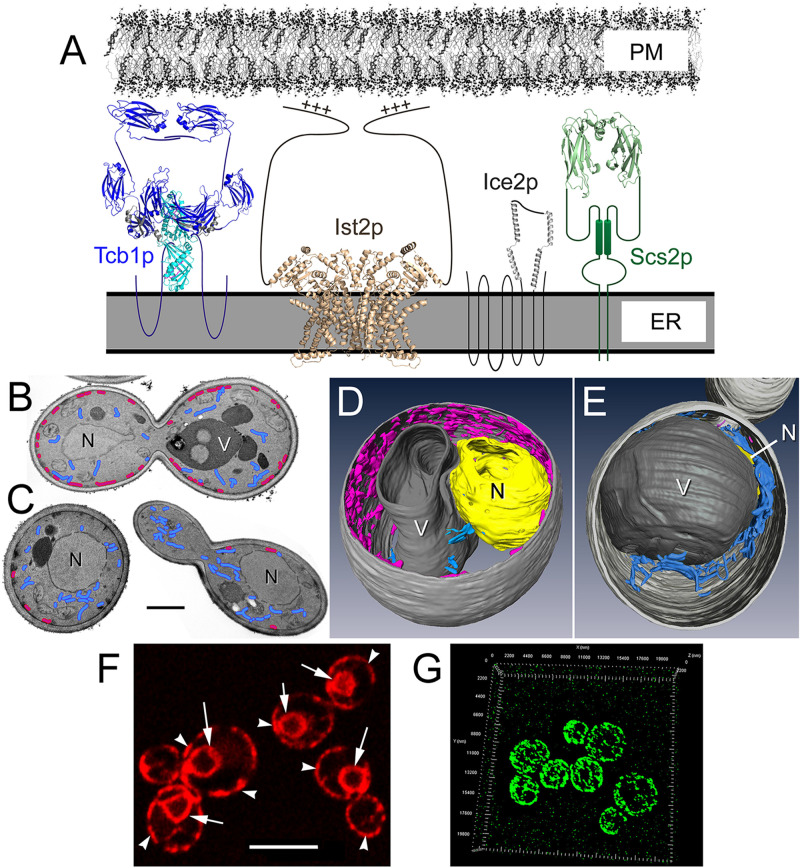
Membrane tether proteins adhere cER membrane to the cytoplasmic face of the PM. **(A)** Representations of the four classes of yeast primary membrane tether proteins that establish ER-PM MCSs, including: a yeast homolog of E-Syts (Tcb1p); the yeast Anoctamin/TMEM16 homolog (Ist2p); the yeast-specific tether Ice2p; and a homolog of VAP (Scs2p). Structures shown are based on homologous proteins, and unstructured elements are represented as simple lines. The Tcb1p dimer representation is shown in the “tunnel model” configuration (Schauder et al., 2014), and structural elements are based on the mammalian E-Syt2 SMP-C2a-C2b domain (PBD ID: 4P42 and 2DMG); C2 domains are dark blue (Tcb1p has 4 C2 domains where E-Syt2 has 3 as shown) and SMP domains are light blue. The Ist2p dimer is based on the TMEM16 lipid scramblase structure PBD ID: 4WIT with the PM-binding C-terminal with the polybasic region indicated (+++). The structure for Ice2p has not been determined though based on its four predicted helical segments and the length of the third cytoplasmic loop, it could extend ∼10 nm from the ER membrane. The Scs2p dimer structure is represented by the VAP-A MSP homology domain (PBD ID: 1Z9L). Estimated model sizes are to scale with the ER and PM shown ∼20 nm apart. **(B)** Representative transmission electron microscopy (TEM) images of wild-type yeast and **(C)** Δ-s-tether cells showing cER (magenta) distribution along the PM and cytoplasmic ER (blue). Only small regions of ER-PM association persist in Δ-s-tether cells. N, nucleus; V, vacuole. Bar = 1 μm. **(D)** Representative wild-type and **(E)** Δ-s-tether cells modeled from 3-D constructions of sections imaged by focused-ion beam scanning electron microscopy (FIB-SEM); cER is shown in magenta, cytoplasmic ER in blue. V, vacuole; N, nucleus (yellow; in the Δ-s-tether cell the nucleus is partially hidden underneath the vacuole). **(F)** RFP-ER fluorescence marks normal nuclear (arrows), cytoplasmic, and cER (arrow heads). Bar = 5 μm. **(G)** 3-D representation of super-resolution micrograph image stacks showing Tcb3p-GFP cortical fluorescence at ER-PM MCSs.

Membrane Contact Sites are regions of close apposition between two organelles that serve as interfaces for both direct exchanges of membrane constituents and coordinating regulatory interactions. MCSs are found in all eukaryotic cells and involve nearly all membrane compartments (Porter and Palade, 1957; Henkart et al., 1976; Manford et al., 2012; Toulmay and Prinz, 2012; Lackner et al., 2013; Hönscher and Ungermann, 2014; Henne et al., 2015; Lang et al., 2015; Saheki and De Camilli, 2017a; Quon et al., 2018; Prinz et al., 2020). A wide variety of tethering proteins bridge the gaps between membranes as exemplified by the contact sites between cortical ER and the PM (Figure 1).

The membrane morphology and association between cER and PM varies greatly among eukaryotic organisms and among different cell types. In yeast, a particularly large proportion of the cytoplasmic face of the PM (∼40%) is covered by closely associated ER membrane (Figure 1; Schuck et al., 2009; West et al., 2011; Quon et al., 2018). In the model plant *Arabidopsis thaliana*, the extent of coverage over the inner surface of the PM by cER is ∼5−10% depending on developmental stage (McFarlane et al., 2017). Lastly, in mammalian cells, the extent of cER coverage over the PM tends to be less and generally ranges from ∼0.25−8%, depending on cell type (Orci et al., 2009; Chen et al., 2019).

The distribution of cER along the PM can also vary in different polarized cell types. For instance, PM-associated cER is more abundant on basal membranes in HeLa cells though in Sertoli cells and polarized epithelia, ER-PM contacts occur at apical membranes (Orci et al., 2009; Son et al., 2016; Lyon et al., 2017). In neuron cell bodies the cER covers 12.5% of the PM inner surface, though only 0.5−1% of the PM contacts ER in axons (Wu et al., 2017; Chen et al., 2019).

In general, the cER associated with the PM in mammalian cells is mainly ribosome free, at least on the ER facing the cell surface, and the cER cisternae exhibits two thicknesses (Smith and Sjostrand, 1961; Orci et al., 2009; Wu et al., 2017). Most cER has a luminal width >25 nm, but “thin ER” can have almost no luminal space. Thin ER expands in cells that overexpress regulatory elements of intracellular Ca^2+^ storage, but Ca^2+^ depletion in generally induces cER association with the PM by several fold (Orci et al., 2009). Given inherent differences in cellular Ca^2+^ regulation and storage in *Saccharomyces cerevisiae* versus metazoans, the dependency of Ca^2+^ homeostasis on yeast ER-PM contact is less significant (Strayle et al., 1999; Cunningham, 2011). Although this review provides a concise description of Ca^2+^ storage and regulation at ER-PM MCSs, we direct readers to several recent reviews that provide comprehensive detail on store-operated Ca^2+^ entry (SOCE) at ER-PM contacts (Prakriya and Lewis, 2015; Derler et al., 2016; Lewis, 2020; Lopez et al., 2020). Here, we focus on the diversity of cER and PM components that establish ER-PM MCSs, as well as associated factors involved in membrane regulation, lipid biosynthesis and transport.

As a major biosynthetic site for lipids and secretory proteins, the ER represents the source of most membrane components. As the major membrane to which these components are targeted, the PM represents the target destination for most ER-derived membrane components. In yeast, the membrane fraction of ER that biochemically co-purifies with the PM is defined as the PM-associated membrane (PAM) (Pichler et al., 2001). PAMs can be biochemically defined as the membrane attached at ER-PM MCSs that is highly enriched in PS, PI, and ergosterol biosynthetic enzymes. The presence of these enzymes suggest that PAMs play an important role in lipid biosynthesis and trafficking between the ER and PM (Pichler et al., 2001). Independent of vesicular transport from the ER, ER-PM MCSs provide another potential “non-vesicular” conduit for lipid transfer critical for maintaining cortical surface area and cell size (Funato et al., 2019). In addition to non-vesicular transport, ER-PM MCSs represent a “sensing nexus” that balances ER metabolic production with the demands of PM expansion during cell growth (Quon et al., 2018). Tether proteins forming ER-PM contact sites mediate unique membrane structures in plants and confer the inheritance of cER into yeast daughter cells during mitosis (Estrada de Martin et al., 2005; Tavassoli et al., 2013; Pérez-Sancho et al., 2016). Lastly, ER-PM contact sites also play important cellular roles in responses to membrane stress (Stefan, 2020). Given the diversity of cell functions affected, it is also not a surprise that ER-PM MCSs defects are implicated in the disease pathology (Nishimura et al., 2004; Fowler et al., 2019).

## Conserved ER-PM MCSs Tether Proteins are Structurally Diverse

The establishment, intermembrane separation, and dynamics of ER-PM MCSs relies on a wide variety of ER-anchored protein tethers that are present at the cER-PM interface. Among most if not all eukaryotes, three major conserved families of ER-PM tethers are represented by VAPs including yeast Scs2p and Scs22p, the E-Syts including plant SYTs and yeast Tricalbins, and members of the Anoctamin/TMEM16/Ist2p proteins (Figure 1A). These families of tethers embody distinct modes of tethering involving either direct links between the ER and PM or multi-subunit complex bridges that connect closely apposed membranes.

### E-Syts Are Both Tethers and Lipid Transfer Proteins

The E-Syts represent tethers that intrinsically span the gap between membranes, potentially forming an intermembrane lipophilic channel (Schauder et al., 2014). The mammalian E-Syts, plant SYT proteins, and the homologous yeast Tricalbins generally contain three regions: (i) an N-terminal hairpin (or, for Arabidopsis SYTs, a type I transmembrane domain (Yamazaki et al., 2010), which inserts into the cytoplasmic leaflet of the ER; (ii) single or multiple SMP (synaptotagmin-like mitochondrial lipid-binding protein) domains that share the physical attributes of TULIP domains containing deep hydrophobic channels capable of lipid binding; and (iii) a variable number of C-terminal C2 domains, which directly bind the opposing PM (Craxton, 2004; Groer et al., 2008; Kopec et al., 2010; Toulmay and Prinz, 2012; Giordano et al., 2013; Pérez-Sancho et al., 2016; Reinisch and De Camilli, 2016). C2 domain interactions with membranes are often potentiated by cytosolic Ca^2+^ and/or facilitated by the presence of phospholipids, such as PI(4,5)P_2_ (Rizo and Südhof, 1998; Chang et al., 2013; Giordano et al., 2013; Lee et al., 2019).

E-Syts are implicated in the possible Ca^2+^-dependent non-vesicular transfer of neutral glycophospholipids, such as DAG, between membranes and two different structural models have been proposed to explain this process (Saheki et al., 2016; Yu et al., 2016). The “tunnel model” proposes that E-Syts facilitate direct lipid transfer between membranes by forming a hydrophobic tunnel between membranes (Schauder et al., 2014). In this model, the E-Syt SMP domains dimerize to form a long singular channel that binds lipids. Although this model is supported by the observed perpendicular orientation of E-Syts at contact sites between the ER and PM, the length of the SMP dimer tunnel (∼9 nm) is less than the observed distances between the ER and PM (∼19–24 nm; Fernández-Busnadiego et al., 2015; Collado et al., 2019); seemingly too short to span between the ER and PM. However, a presumed obligatory intermembrane distance is a somewhat contrived concept, and only relevant if the lipid tunnel model does not faithfully represent the in vivo conformation of E-Syts (Saheki and De Camilli, 2017a; West et al., 2011; Schauder et al., 2014; Quon and Beh, 2015; Reinisch and De Camilli, 2016; Saheki and De Camilli, 2017a; Saheki and De Camilli, 2017b; Quon and Beh, 2015; Schauder et al., 2014; Reinisch and De Camilli, 2016). Alternatively, in the “shuttle model,” E-Syts act as membrane-anchored lipid transfer proteins that bind and move lipids between the two closely aligned ER and PM bilayers (Schauder et al., 2014; Bian et al., 2019). However, these two structural options might confer different functions and are not necessarily mutually exclusive (Collado et al., 2019).

In mammalian cells, E-Syt2 and E-Syt3 contain an SMP domain followed by three C2 domains whereas E-Syt1 contains five C2 domains (Giordano et al., 2013; Reinisch and De Camilli, 2016; Saheki and De Camilli, 2017b). Previous work has shown that E-Syt2 and E-Syt3 form ER-PM contacts independent of Ca^2+^ while E-Syt1 is recruited to the contact sites when Ca^2+^ levels rise (Giordano et al., 2013; Fernández-Busnadiego et al., 2015; Idevall-Hagren et al., 2015; Yu et al., 2016). Hence, ER-PM junctions are dynamically regulated by Ca^2+^ signaling. In fact, increased Ca^2+^ levels not only promote E-Syt1-mediated ER-PM association in mammalian cells, but also shortens the distance between the ER and the PM and activates SMP-dependent lipid transfer (Fernández-Busnadiego et al., 2015; Saheki et al., 2016; Bian et al., 2018). At the “resting state,” a small pool of E-Syt1 resides at ER-PM contact sites in the absence of Ca^2+^ and the distance between the ER and the PM at these sites is larger than E-Syt2 or E-Syt3 associated ER-PM contact sites (Fernández-Busnadiego et al., 2015; Pérez-Lara and Jahn, 2015). This difference in distance is likely due to different chain length of cytosolic segments of E-Syt1 and E-Syt2/3, given that they contain different numbers of C2 domain units. E-Syt1 C2E domain is highly similar to C2C E-Syt2/3 domain, which enables constitutive interaction to PM in the normal conditions (Fernández-Busnadiego et al., 2015). As Ca^2+^ levels increase, the E-Syt1 C2C domains interact with the PM to lessen the ER-PM gap (Fernández-Busnadiego et al., 2015).

The Ca^2+^ dependency of the yeast Tricalbin E-Syt homologs and plant SYTs is less clear. Recruitment of the Tricalbins and SYTs is Ca^2+^ independent, but Ca^2+^ does play a role both in increasing SYT phospholipid binding affinity (Schapire et al., 2008), and shortening Tcb3p-mediated ER-PM MCS distances (Hoffmann et al., 2019). At sites of Tcb3p-mediated cER-PM contact, high cytosolic Ca^2+^ shortened the distance between ER and PM by ∼2 nm (Hoffmann et al., 2019). This observation might indicate that the shortening of the SMP-C2 bridge at these sites resulted in more C2 proteins coating the PM than in wild-type cells. A denser protein coat across the PM was observed under these conditions. An intermediate density is also associated with E-Syt ER-PM contact sites (Fernández-Busnadiego et al., 2015).

### VAP Homologs Are Scaffolds for Tether Complexes

The VAPs represent a different mechanism in membrane tethering. Given that they do not contain any domain capable of making contact *in trans* with an opposing membrane, VAPs necessarily form multi-subunit bridges. The domain architecture of eukaryotic VAPs, including yeast Scs2p/22p, consists of three major regions: (i) a MSP domain, which includes the binding site for proteins containing the FFAT (“two phenylalanines in an acidic tract”) motif (Loewen et al., 2003); (ii) a single C-Terminal ER transmembrane domain; (iii) an intermediate variable linker region, which is ∼27 nm in VAP-A and Scs2p (Loewen et al., 2003, 2007; Loewen and Levine, 2005; Murphy and Levine, 2016). Scs2p and Scs22p have both been shown to bind phosphoinositides, such as PI4P, through their N-terminal MSP domain which extends out toward to PM (Kagiwada and Hashimoto, 2007). Considering that the linker region length between the transmembrane domain and the MSP domain in Scs2p could conceivably span the two membranes, it is tempting to postulate that Scs2p directly associates the two membranes. However, Scs2p or Scs22p have not been demonstrated to directly interact with the PM. Furthermore, mutations in the MSP domain that disrupt FFAT motif binding inhibit Scs2p-dependent ER-PM membrane association, suggesting that FFAT protein-MSP domain interactions are required for contact (Manford et al., 2012).

In terms of promoting ER-PM MCSs, tether proteins are not functionally equivalent. In yeast, the elimination of Scs2p (the VAP-B homolog) and its paralog Scs22p (most homologous to VAP-A) results in a ∼50% reduction in ER-PM association within yeast cells, which is unmatched in its severity when compared to the removal of any other tether protein (Loewen et al., 2003, 2007; Manford et al., 2012). The many functional and physical interactions involving VAPs and Scs2p (and likely Scs22p also) suggest they act as scaffolding proteins to assemble additional subunits at MCSs to physically connect membranes (Murphy and Levine, 2016). Unlike Scs2p, however, the function of Scs22p is more ambiguous. Within Scs2p, a linker region of ∼20 nm separates the MSP and transmembrane domains, which contributes to the overall distance Scs2p can span between the ER and PM (Murphy and Levine, 2016). Within Scs22p, this linker region is all but absent (Loewen and Levine, 2005), though it is unclear if this segment is even relevant to a multi-subunit intermembrane bridge. It should be noted that Scs22p and Scs2p are not equivalent as tethers, though there appears to be a minor functional overlap (Craven and Petes, 2001; Loewen and Levine, 2005). Scs2p and Scs22p might share specific interactions with some lipid transfer proteins (Riekhof et al., 2014; Weber-Boyvat et al., 2015). However, if Scs2p and Scs22p have shared roles in membrane tethering, it is not manifested in any observed differences in cER-PM association when comparing *scs2*Δ and *scs2*Δ *scs22*Δ cells (Loewen et al., 2007; Manford et al., 2012). Although *SCS22* is routinely deleted along with *SCS2* to remove all yeast VAP activity, the specific *in vivo* functions of Scs22p deserve additional attention.

Arabidopsis VAP27 proteins represent plant homologs of both mammalian VAPs and yeast Scs2p (Sutter et al., 2006; Saravanan et al., 2009). The 10 identified VAP27 proteins in *A. thaliana* (VAP27-1 to VAP27-10) can be grouped into 3 clades (Wang et al., 2016). Members of two of the clades have a single TM domain that determines their localization to ER, whereas members of the third lack the TM domain, and are localized to the PM (Wang et al., 2016). The best studied of these plant proteins is VAP27-1, which consists of a C-terminal ER-spanning transmembrane domain, a coiled-coil domain, and the MSP domain (Wang et al., 2014, 2016). Although VAP27-1 can bind phosphoinositides *in vitro*, VAP27-1 likely forms an ER-PM bridge in a protein complex with NET3C, which belongs to the plant-specific NET proteins that link actin filaments to different endomembranes within the cell (Deeks et al., 2012; Wang et al., 2014, 2016; Stefano et al., 2018). As a multi-subunit tether complex, VAP27-1 and NET3C form ER-PM MCSs, in which NET3C also binds actin filaments and VAP27-1 might bind microtubules (Wang et al., 2014; Siao et al., 2016; Stefano et al., 2018). However, VAP27-1/NET3C MCSs are not affected by cytoskeletal disruption, even though *vap27-1 vap27-3* double mutant plants exhibit a disrupted ER and actin morphology, which is linked to growth aberrant establishment of cell polarization (Wang et al., 2016; Stefano et al., 2018).

### Specific Anoctamin/TMEM16/Ist2p Homologs Are ER-PM Tethers

The structural architecture of Anoctamin/TMEM16/Ist2p homologs is unique among membrane proteins (Brunner et al., 2016). They have multiple transmembrane helices forming dimers embedded within membranes. Between subunits, a cavity forms at dimeric interfaces and it is potentially filled with lipids. In the PM or ER, this dimer cavity provides a possible passage route for lipids from one leaflet of the bilayer to the other. This lipid scramblase activity is one major function ascribed to Anoctamin/TMEM16/Ist2p homologs, but some act as Cl^–^ ion transporters, while others have one or none of the activities (Brunner et al., 2016; Whitlock and Hartzell, 2017). A select number of these proteins are found at ER-PM MCSs.

The yeast TMEM16 homolog, Ist2p is a primary tether that has extended sequences long enough to bridge the gap between the ER and PM (Maass et al., 2009; Kralt et al., 2015). Ist2p consists of an ER transmembrane region that shares homology with the Anoctamin (ANO/TMEM16) protein family, a C-terminal cortical sorting sequence, and a cytosolic inter-membrane linker. The cortical sorting sequence is a lysine-rich domain of low complexity that mediates direct PM binding by interacting with PI(4,5)P_2_ (Ercan et al., 2009; Fischer et al., 2009; Maass et al., 2009; Wolf et al., 2012). Ist2p plays a major role at PM-ER MCSs because the deletion of *IST2* alone causes a significant reduction in membrane association (Manford et al., 2012; Wolf et al., 2012). Indeed, in scs2Δ scs22Δ cells, the additional deletion of IST2 has a greater impact on cER-PM association than deleting all three Tricalbins (Manford et al., 2012). Unlike the Tricalbins and Scs2/22p, the domain of Ist2p that extends from the ER to the PM has little defined structure, but it serves the simple requirement of being long enough to bridge the inter-membrane distance, but it may have another specific function.

The bulk of Ist2p homology to ANO/TMEM16 proteins is located within its multi-pass transmembrane domain region (Ercan et al., 2009; Hartzell et al., 2009). Of the ten human ANO proteins (ANO1-ANO10), Ist2p shares the greatest similarity to ANO10 (TMEM16K) (Kunzelmann et al., 2016). Most of the human ANO proteins, such as ANO1/2 (TMEM16a/b), localize to the PM and act as Ca^2+^-activated Cl^–^ ion channels. Other ANO homologs, such as human ANO6 (TMEM16f) or *A. fumigatus* and *N. haematococca* TMEM16, appear to have phospholipid scramblase activities (Caputo et al., 2008; Schroeder et al., 2008; Yang et al., 2008; Hartzell et al., 2009; Malvezzi et al., 2013; Brunner et al., 2014; Yu et al., 2015). However, when directly assayed Ist2p does not seem to have lipid scramblase activity (Malvezzi et al., 2013). Because Ist2p shares a similar Ca^2+^-dependent sorting pathway to the cER with the mammalian ER Ca^2+^ sensor stromal interaction molecule 1 (STIM1), it has been proposed that Ist2p might be a functional equivalent of STIM1 for yeast regulation of cytoplasmic Ca^2+^ (Kunzelmann et al., 2016; Wanitchakool et al., 2017). If Ist2p or other ER-PM tethers play a role in yeast Ca^2+^ regulation, one might predict that eliminating these proteins would affect Ca^2+^ homeostasis. However, *Saccharomyces cerevisiae* lacks SERCA (sarco/endoplasmic reticulum Ca^2+^-ATPase)-family Ca^2+^ transporters and both the yeast Golgi and vacuole are the major Ca^2+^ storage organelles, not the ER as in metazoans (Strayle et al., 1999; Cunningham, 2011). Thus, the budding yeast ER and ER-PM MCSs seemingly play a lesser role in affecting intracellular Ca^2+^.

### Membrane Structure at Yeast ER-PM MCSs Is Shaped Differently by Each Tether

In terms of basic protein structure, Ist2p and all yeast tethers are ER integral membrane proteins (Figure 1A). However, only Ist2p and the Tricalbins are predicted to directly span between cER and PM to make contact. In contrast, Scs2p/22p lack obvious PM interaction domains, and it is unclear if the yeast-specific tether Ice2p (see below) has cytoplasmic domains long enough to link the ER to the PM (Figure 1A). To provide membrane attachment, these proteins would seem to require additional binding subunits. Bridging between membranes through an extended protein complex is an established mode of membrane tethering. In the multi-subunit ER-mitochondria encounter structure (ERMES), ER-mitochondria contact requires several subunits (Lang et al., 2015). In a different context, the multi-subunit exocyst complex also tethers vesicle membranes to the PM during the last steps of exocytosis (Lepore et al., 2018). Whether a single intrinsic tether, or a multi-subunit bridge complex, the varied mechanisms of tethering defies simplistic definitions as involving only a single protein that spans between closely apposed membranes.

In yeast, the distance between cER and the PM is variable (∼16 to 59 nm; West et al., 2011), therefore no absolute domain length is required for a tether to reach between membranes. In fact, the transverse distance between the cER and PM appears to be highly dynamic and morphologically adaptable (Collado et al., 2019). In yeast, an artificially constructed ER-PM staple can functionally complement growth defects in cells lacking ER-PM tethers, despite being predicted to extend between membranes by only ∼10 nm (Quon et al., 2018). Therefore, estimated *trans*-membrane linker length does not necessarily reflect tethering function of which some can be conferred by non-specific ER-PM physical contact.

Analysis of ER-PM MCSs by cryo-electron tomography and correlative light and electron microscopy has shown that the association of different tether protein families with the PM involves distinct ER shapes (Fernández-Busnadiego et al., 2015; Saheki et al., 2016; Collado et al., 2019; Hoffmann et al., 2019). In wild-type cells, cER has regions that lay flat across the PM with other regions where the ER is tubular (West et al., 2011; Nixon-Abell et al., 2016). In cells lacking six primary tethers, expression of the yeast anoctamin/TMEM16 homolog Ist2p generates cER membrane that is tubular and sheet-like, similar to wild-type cER in width and distance from the PM (Collado et al., 2019). This result suggests that Ist2p plays an important role in shaping cER morphology (Collado et al., 2019). When Scs2p/Scs22p are expressed in cells lacking many ER-PM tethers, cER morphology is significantly altered and is mostly extended ER sheets that are narrow in width, though the distance of cER to the PM is unaffected (Collado et al., 2019). Scs2p/Scs22p appears important for maintaining ER width and does not determine cER-PM distance. The expression of only the yeast E-Syt homologs, the Tricalbins Tcb1p/Tcb2p/Tcb3p, in cells lacking other tethers resulted in cER with mainly tubular structure, and the ER membrane curves toward the PM (Collado et al., 2019; Hoffmann et al., 2019). This curvature brings ER in very close proximity to the PM, where the cER forms peaks of radius ∼10 nm at the base and height ∼7 nm.

Because ER tubulation is facilitated by reticulons, it is surprising that Tricalbin-generated ER curvature is reticulon-independent (Hoffmann et al., 2019). In the absence of reticulon-like proteins Yop1p and Rtn1p the ER loses its tubular structure and expands, but Tcb3p still forms highly curved tubules at ER-PM MCSs (Voeltz et al., 2006; West et al., 2011; Hoffmann et al., 2019). An *in situ* structural analysis of Tricalbins showed that rod-shaped structures, comprising SMP and C2 domains, connect the ER to the PM where Tcb3p seems to occupy a dense coat along the PM (Hoffmann et al., 2019). These results indicate that tethers not only link the ER and PM together, but in doing so they alter local membrane structure around contact sites.

## Mammalian ER-PM MCSs Mediate Ca^2+^ Regulation

Mammalian cells rely on ER-PM MCSs to regulate and maintain intracellular levels of Ca^2+^. In metazoan cells, ER-PM MCSs regulate intracellular Ca^2+^ levels through SOCE that refills ER Ca^2+^ stores via SERCA pumps (Liou et al., 2005; Zhang et al., 2005; Feske et al., 2006; Wu et al., 2006; Anderie et al., 2007; Lewis, 2007; Luik et al., 2008; Hogan et al., 2010; Manjarrés et al., 2010; Zhou et al., 2010; Haj et al., 2012). Different isoforms of SERCA pumps, SERCA2 and SERCA3, associate with STIM1 and function in SOCE (Jousset et al., 2007; Lopez et al., 2008; Sampieri et al., 2009). Whereas SERCA2 has higher affinity for Ca^2+^ binding and it is dependent on actin filaments, SERCA3 is more prominent in human platelets and operates independently from the actin cytoskeleton, which indicates two distinct modes of SOCE regulation (Rosado et al., 2004; Lopez et al., 2005, 2006). During SOCE, Ca^2+^ depletion in the ER triggers oligomerization of the ER protein STIM1, which reaches across the ER and PM to bind and activate the Ca^2+^ channel Orai1 at the PM (Luik et al., 2006, 2008). Orai1 activation then drives an influx of extracellular Ca^2+^ for ER replenishment. In this regard, STIM1 and Orai1 represent a Ca^2+^-regulated ER-PM tether complex. Upon Ca^2+^ depletion, cells overexpressing STIM1 induce greater cER in association with the PM (Orci et al., 2009). As with other membrane tethers, ancillary proteins regulate the stability of membrane connections. The ER-resident membrane protein TMEM110/STIMATE regulates, in part, the dynamic changes in STIM-Orai1 MCSs during store-dependent calcium signaling (Quintana et al., 2015).

STIM1 ER-PM contacts are structurally different from those mediated by E-Syts (Fernández-Busnadiego et al., 2015). Unlike E-Syts, STIM1 tethers the ER to the Orai Ca^2+^ channels in the PM upon reduction of ER luminal Ca^2+^ (Carrasco and Meyer, 2011). STIM1-mediated ER-PM contact sites do not form dense layer across the PM, which is in contrast to those at E-Syt mediated ER-PM contact sites (Fernández-Busnadiego et al., 2015). The role of Ca^2+^ at E-Syt MCSs is also different. Although C2 domains, such as those in the three mammalian E-Syts, are often associated with Ca^2+^-dependent membrane interactions, only E-Syt1 seems affected by intracellular Ca^2+^ levels (Giordano et al., 2013). E-Syt1 is diffusely localized throughout the ER under normal growth conditions, but its cortical localization is significantly enhanced in response to high intracellular Ca^2+^ levels associated with SOCE. In contrast, E-Syt2 and E-Syt3 mediate constitutive PM-ER membrane tethering irrespective of Ca^2+^ levels (Chang et al., 2013; Giordano et al., 2013; Fernández-Busnadiego et al., 2015; Idevall-Hagren et al., 2015; Yu et al., 2016). Even though E-Syt1 appears to be affected by SOCE, E-Syt1 plays no detectable role in SOCE regulation suggesting it represents a functionally different class of tethers from STIM/Orai (Giordano et al., 2013). Curiously, even though E-Syt1 can form heterodimers with E-Syt2 and 3, E-Syt1 exhibits a unique ER localization under normal conditions (Giordano et al., 2013; Saheki et al., 2016). It is unclear whether different heterodimeric combinations enable E-Syts to fulfill a broader spectrum of functional roles or to respond differentially to cellular stimuli. As in yeast, however, metazoan E-Syts are likely to be functionally redundant with other membrane tethers given that mice lacking all three E-Syts are normal (Schauder et al., 2014; Tremblay and Moss, 2016).

Neuronal cells also contain long regions of ER-PM contact, including associations mediated by ER tubules (Fernández-Busnadiego et al., 2015; Chang et al., 2017; Wu et al., 2017; Fowler et al., 2019). Even though the distance between the ER and the PM at contact sites are similar to other mammalian cells, neuronal cell bodies contain more contact sites (Fernández-Busnadiego et al., 2015; Wu et al., 2017). Although E-Syts are ubiquitously expressed, they are enriched in the brain. In this tissue, E-Syts might promote more efficient lipid transport within neuronal processes where, due to their long length, the efficiency of vesicular transport might be limited (Min et al., 2007).

Additional ER-PM tethers also participate in neuronal excitation through clustering and integrating ion channel activation. For instance, in mammalian striatal MSNs, PM Kv2.1 voltage-gated potassium channels and ER RyR Ca^2+^ channels are juxtaposed and cluster at ER-PM MCSs, which couples Ca^2+^ release with Kv2.1 regulation (Mandikian et al., 2014). Kv2.1 organizes and remodels ER-PM MCSs through direct FFAT motif-dependent interactions with VAP-A and VAP-B (Johnson et al., 2018; Kirmiz et al., 2018a, b). In addition to VAP recruitment at these ER-PM sites, Kv2.1 channels organize both PM-localized LTCC and the ER-localized RyR Ca^2+^ channels (Vierra et al., 2019). Together Kv2.1, LTCCs and RyR provide a platform for localized Ca^2+^ sparks in soma and proximal dendrites of brain neurons, independent of action potentials (Vierra et al., 2019). Kv2.1 accumulation at ER-PM contact sites is limited by VAP-A and VAP-B recruitment suggesting the functional coupling of LTCCs and RyRs in Ca^2+^ release is indirectly controlled by VAPs. However, a detailed mechanism for how these proteins are interconnected at ER-PM contact sites is still to be established.

## ER-PM Tethers in Budding Yeast are Mechanistically Varied and Functionally Non-Specific

In yeast, the physical attachment between the cER and the PM is mediated by Scs2p, Scs22p, the Tricalbins Tcb1p-3p, Ist2p, as well as the yeast-specific ER integral membrane protein Ice2p (Estrada de Martin et al., 2005; Loewen et al., 2007; Toulmay and Prinz, 2012; Wolf et al., 2012; Quon et al., 2018; Figure 1A). These ER-integral membrane proteins interact with the cytoplasmic face of the PM. For some of these tether proteins, the precise mechanism for PM binding has yet to be determined, though under standard growing conditions all are functionally required for ER-PM association (Quon and Beh, 2015; Quon et al., 2018).

Six ER-PM tether proteins were identified by Manford et al. (2012), who reasoned that tethers might directly interact with both the PI4P phosphatase Sac1p and the VAP-B homolog, Scs2p. In yeast, Sac1p is an integral membrane protein restricted to the ER that may or may not regulate PM PI4P levels *in trans* at regions of close ER-PM apposition (Manford et al., 2010; Stefan et al., 2011; Zewe et al., 2018).

In wild-type cells, PI4P is concentrated in the Golgi, exocytic vesicles, and at the PM near sites of polarized growth at the bud tip. However, the elimination of Sac1p dramatically affects PI4P in the PM, despite the restricted localization of Sac1p in the ER, and under some conditions, the Golgi (Stefan et al., 2011; Piao and Mayinger, 2012; Venditti et al., 2019). In *SAC1* deletion mutants, PI4P accumulates in the PM surrounding both the mother and daughter bud. In cells lacking both *SCS2* and its homolog *SCS22*, a more modest but similar redistribution of PI4P in the PM was also noted (Manford et al., 2012). These results suggested, that Sac1p might act on PI4P in the PM *in trans* from sites associated where cER and PM meet. On the premise that unidentified tethers would be at these contact sites alongside Scs2p and Sac1p, where they might physically interact, a proteomic strategy was successfully applied to discover additional ER-PM tether proteins (Manford et al., 2012).

From the list of Sac1p- and Scs2p-interacting proteins generated by proteomic analysis, the potential tethers identified included three Tricalbins and Ist2p (Manford et al., 2012; Saheki and De Camilli, 2017b). As these integral membrane proteins had been independently found to reside at cER-PM MCSs, this proteomic approach was validated (Toulmay and Prinz, 2012; Wolf et al., 2012). Although not further investigated, many other Sac1p-Scs2p interacting proteins might also fulfill important roles at ER-PM MCSs.

Given their interaction with both Sac1p and Scs2p at ER-PM MCSs, Ist2p and the Tricalbins could conceivably form one large protein complex with coupled functionalities. Despite their interactions with both Sac1p and Scs2p, these tether proteins represent independent MCS complexes with distinct functional roles. Live cell fluorescence microscopy of yeast revealed that the distribution of different tether protein families at ER-PM MCSs do not completely overlap (Toulmay and Prinz, 2011; Manford et al., 2012; Hoffmann et al., 2019). Regardless, in yeast cells the elimination of these tether complexes results in a drastic reduction in ER-PM MCSs, and defects in PI4P regulation approaching that in *sac1*Δ cells (Manford et al., 2012). Recent evidence suggests that E-Syt2 may also regulate PI4P by recruiting the mammalian Sac1 to the cortex and modulating its dynamic localization (Dickson et al., 2016). Considering that the tether proteins in yeast were in part discovered though their physical interactions with Sac1p, this dynamic interaction might represent an important function of E-Syts/Tricalbins (Manford et al., 2012).

Although ER-PM association is greatly reduced in cells lacking six primary tethers (Scs2p, Scs22p, Tcb1p-3p, and Ist2p), ER-PM association is not entirely eliminated (Manford et al., 2012). Even if all ER-PM tether proteins were eliminated, Brownian motion would be predicted to generate random interactions between untethered ER and the PM. As ER diffuses through the cytoplasm near the vicinity of cell cortex, stochastic associations of freed ER might be expected. Through simple volumetric calculations, rough estimates can be made of residual random ER-PM associations remaining in cells lacking tethering proteins.^1^ Even so, in cells lacking just the six tether proteins, the coverage of PM with ER is still several fold more than the stochastic estimate. This calculation suggested that there is at least one more primary tether protein.

### Ice2p Represents a Yeast-Specific ER-PM Tether Required for Organelle Inheritance

Ice2p is an ER Type III transmembrane protein, which also contributes to ER-PM tethering (Quon et al., 2018). Ice2p facilitates the movement and inheritance of cER along the PM from yeast mother cells into daughter cells (Estrada de Martin et al., 2005; Loewen et al., 2007). Yeast cER inheritance involves myosin-directed movement of cER tubules along the PM into emerging daughter buds via actin cables positioned parallel to the mother-daughter axis (Du et al., 2001).

Apart from its role in cER inheritance, Ice2p is recognized for multiple events in inter-organelle contact (Estrada de Martin et al., 2005; Loewen et al., 2007; Toulmay and Prinz, 2011; Tavassoli et al., 2013; Markgraf et al., 2014; Rogers et al., 2014; Quon et al., 2018). Ice2p plays distinct roles in organelle associations depending on cellular growth phase. When cells exit from stationary phase, Ice2p facilitates a direct physical interaction between the ER and lipid storage droplets, where it regulates the flow of neutral lipids from lipid droplets into the ER (Markgraf et al., 2014). As cells re-enter exponential growth, Ice2p rapidly re-localizes to the cER at punctate sites along the PM (Estrada de Martin et al., 2005; Markgraf et al., 2014; Quon et al., 2018). The deletion of *ICE2* in *scs2*Δ cells also has considerable impact on mitotic cell growth and PC synthesis (Loewen et al., 2007; Tavassoli et al., 2013). These results are consistent with Ice2p being physically located at ER-PM MCSs and ER-lipid droplet contacts, depending on growth state.

Based on possible secondary structure predictions, Ice2p likely contains no large cytoplasmic domain or defined lipid binding domains capable of bridging across membrane gaps (Markgraf et al., 2014). Nonetheless, the third cytoplasmic loop of Ice2p is predicted to have four amphipathic helices with unstructured linker regions that might in fact extend between membranes at particularly close distances. This third Ice2p loop appears to be particularly important for its linking to LDs, and when expressed alone the Ice2p cytoplasmic loop does bind LDs and to a lesser degree the ER (Markgraf et al., 2014). Whether this loop is also pertinent for ER-PM association remains to be seen, though its estimated length is just under the smallest observed distance (16 nm) between cER and the PM (West et al., 2011).

By deleting *ICE2* in the strain lacking the six other tethers, Δ-s-tether cells were generated in which ER-PM association is further reduced to calculated levels of random/stochastic contact (Quon et al., 2018; Figures 1B−E). Although in Δ-s-tether cells ER-PM membrane contact is all but eliminated, these cells are viable and grow slowly, at least under standard growth conditions. The deletion of *ICE2* not only reduces cER-PM association beyond that conferred by eliminating the other six tethers, but it also significantly exacerbates phospholipid regulatory defects (Quon et al., 2018). In Δ-s-tether cells, phospholipid levels (PE, PC, and PA) are substantially reduced as are sphingolipid levels, whereas DAG levels are elevated. The addition of the PC precursor choline rescues Δ-s-tether growth defects, suggesting that a major function of yeast ER-PM MCSs involves the regulation of phospholipid synthesis. Curiously, sterol levels are unaffected in Δ-s-tether cells, even though sterol depletion causes cER expansion to nearly encompass the entire PM (Quon et al., 2018). Perhaps the assembly of ER-PM MCSs is regulated by membrane structure changes or stresses, as triggered by sterol depletion, which also affects phospholipid composition.

### Ancillary Regulators and Effectors Also Contribute to ER-PM MCSs Function in Yeast

At pre-established MCSs, secondary tethers and ancillary regulators might induce contact in response to specific stimuli or might fortify and expand existing membrane association. As opposed to primary tethers required for ER-PM contact under standard conditions, secondary tethers would be sufficient (but not necessary) to promote membrane association when overexpressed or induced (Quon and Beh, 2015). Ancillary regulators include those that affect ER structure and ER shape, which influences cER association with the PM and other membrane organelles. Examples of conserved and cell type-specific modulators of ER morphology include: the dynamin-like GTPase atlastin-1 (Sey1p in yeast), reticulons, the ER/Ca^2+^/synaptic vesicle regulator secernin-1, and “receptor expression-enhancing protein” (REEP; homologs of yeast reticulon-interacting protein Yop1) (Park et al., 2010; Beetz et al., 2013). Several of these proteins sculpt ER shape through interactions with cytoskeletal elements such as microtubules (Park et al., 2010; Lindhout et al., 2019).

In addition to cytoskeletal interactions, many ER-PM MCS effectors make direct contact with tether proteins. Around half of VAPs/Scs2p/Scs22p interactions involve binding to “FFAT-motif proteins,” including a subset of the family of ORPs homologs (Murphy and Levine, 2016). Despite their homology to the canonical mammalian “OSBP,” not all ORPs actually bind oxysterols or sterols (Pietrangelo and Ridgway, 2018). However, ORPs act as lipid transfer proteins *in vitro* and are implicated in both non-vesicular and vesicular transport pathways *in vivo* (Alfaro et al., 2011; de Saint-Jean et al., 2011; Beh et al., 2012; Mesmin et al., 2013; Chung et al., 2015; Moser von Filseck et al., 2015a, b; Kentala et al., 2016; Tong et al., 2016). Most ORPs in mammals are associated with membrane contact sites between organelles, which appears true for some if not all yeast ORPs homologs as well (Kentala et al., 2016; Pietrangelo and Ridgway, 2018). In yeast, Scs2p binds three ORP homologs (Osh1p-3p) via their FFAT motif, which is found within an N-terminal region aside a PI4P-binding PH domain (Roy and Levine, 2004; Stefan et al., 2011; Tong et al., 2013; Weber-Boyvat et al., 2015). All yeast Osh proteins are soluble but in addition to the cytoplasm, Osh2p and Osh3p can localize to the cell cortex and Osh1p is targeted to sites at the NVJ (Levine and Munro, 2001; Kvam and Goldfarb, 2004). Osh2p and Osh3p localization with cER at ER-PM MCSs is dependent on PI4P and is significantly reduced by repression of Stt4p, the PM PI4P kinase (Stefan et al., 2011). Scs2p/22p binding to FFAT motifs also contribute to Osh2p/3p cortical localization, as well as to Osh1p NVJ localization (Loewen et al., 2003). However, in scs2Δ scs22Δ mutant cells, Osh3p colocalization with cER and PM-ER MCSs is disrupted, though it remains associated with the PM (Stefan et al., 2011). These results suggested that Osh3p acts as a PM-binding adaptor subunit for a Scs2p tethering complex that bridges between the ER and PM (Stefan et al., 2011). However, OSH3 deletion has not been reported to disrupt ER-PM association and Scs2p binding is not required, though localization of Osh3 to the cortex is needed for its functionality (Stefan et al., 2011).

A recent paper reported transient interactions between Osh2p and nascent endocytic sites on the PM near associated “cER rims” (Encinar del Dedo et al., 2017). Through interactions with the type I myosin Myo5p and Scs2p, it is proposed that Osh2p regulates cER-endocytic site associations to facilitate actin patch assembly for endocytic internalization (Encinar del Dedo et al., 2017). These studies raise the possibility that Osh2p and Scs2p couple Myo5p-dependent actin polymerization with the clearance of cER from endocytic sites undergoing membrane invagination. Even in this case, however, there is no evidence that Osh2p provides a stable link for ER-PM contact. Given the lack of functional data, the role of Osh proteins and other FFAT motif proteins in promoting general MCS assembly is still an important but open question.

## The Cellular Coordination of Phospholipid Metabolism and Transport Requires ER-PM MCSs and Lipid Transfer Proteins

**FIGURE 2:**
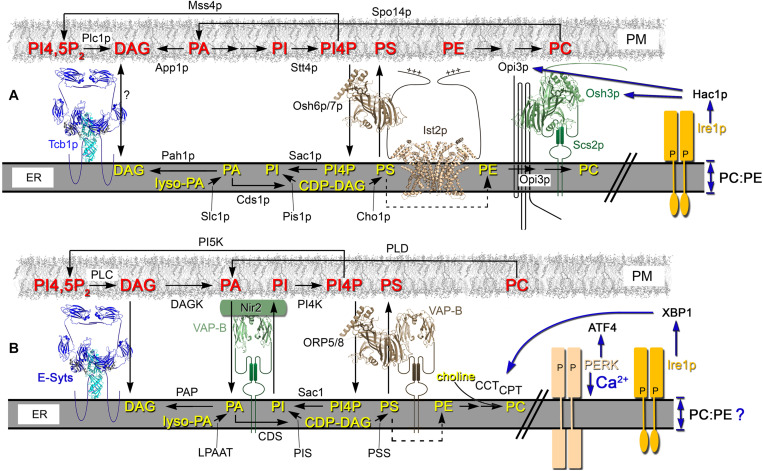
In yeast and mammalian cells, lipid flux through the ER and PM is partly coordinated through ER-PM MCSs. **(A)** In yeast cells, a primary source of PA in the PM involves Spo14p (PLD)-mediated conversion of PC. In the ER, the CDP-DAG pathway also generates PA that generates CDP-DAG, which is a precursor for PI and PS synthesis. Through its association with Ist2p, Osh6p/7p (Osh proteins and ORPs are modeled on the PI4P-bound structure of Osh4p, PBD ID: 3SPW) reciprocally exchanges PS from the ER to PM for PI4P generated in the PM by the PI4 kinase Stt4p. Acting near or in association with ER-PM MCSs, the ER PI4P phosphatase Sac1p hydrolyzes PI4P to PI. In the PM, the deposited PS is used to produce PE that is the precursor for PC synthesis by Opi3p, which interacts with Osh3p and acts *in trans* at ER-PM MCSs. ER bilayer perturbations that reduce the membrane PC:PE ratio activate Ire1p-dependent UPR through the Ire1p transmembrane domain. The Hac1p-dependent transcriptional program induces UPR, which affects the transcription of some lipid biosynthetic enzymes and regulators (e.g., Opi3p, Osh3p [as shown] and also Slc1p) (Travers et al., 2000; Schuck et al., 2009). This transcriptional regulation operates in parallel with the Opi1-regulated pathway of phospholipid biosynthetic gene expression, which directly responds to PA levels (Schuck et al., 2009). **(B)** In mammalian cells, DAG Kinase (DAGK) converts DAG to PA in the PM, which is then transported to the ER via Nir2. Nir2 is a PA/PI exchanger that reciprocally transfers PI from the ER to the PM. Once transferred to the PM, PI is converted to PI4P by PI4 kinase (PI4K) and PI4P can be transported to the ER via VAP-B interacting proteins ORP5/8. In the ER, PI4P is hydrolyzed to PI by the Sac1p phosphoinositide phosphatase. In the PM, PI4P is converted into PI4,5P_2_ by PI5 Kinase (PI5K) and ER-resident tethers, such as the E-Syts, physically interact with PI4,5P_2_ to mediate PM contact. PI4,5P_2_ levels are controlled by phospholipase C (PLC), which converts PI4,5P_2_ into DAG. Excess DAG in the PM is transported to the ER via E-Syt tether proteins. In the ER, lyso-PA is a precursor used by lyso-PA acyltransferase (LPAAT) for PA synthesis, which is the precursor for the production of DAG by phosphatidate phosphatase (PAP) or CDP-DAG by CDP-DAG synthase (CDS). CDP-DAG is a precursor for both PI by PI synthase (PIS) and PS by PS synthase (PSS). Both PI and PS are transported to PM by Nir2 and ORP5/8, respectively. PS in the ER is transported to the mitochondria where it generates PE, which is returned to the ER (dotted arrow). In the ER, PE can be converted to PC by PE N-methyltransferase (PEMT). In the Kennedy pathway, cytosolic CDP-choline generated by CCT and CPT activities produce PC by combining choline with DAG in the ER. Vesicular transport carries PC generated in the ER to the PM. In mammalian cells, the transmembrane domain of both Ire1 and PERK are important for UPR signaling as sensors of lipid perturbations and Ca^2+^ depletion (Volmer et al., 2013). PC synthesis is enhanced by UPR activation by increasing CCT and CPT biosynthetic activities in a post-transcriptional manner (Sriburi et al., 2004).

The function of ER-PM MCSs is tightly coordinated with phospholipid biosynthesis (Figure 2; Toulmay and Prinz, 2011; Stefan et al., 2013; Omnus et al., 2016; Quon et al., 2018). The synthesis of all major membrane phospholipids begins with PA (Carman and Henry, 1999, 2007). In yeast cells, PA is produced either from Lyso-PA in the ER or from DAG in the PM (Figure 2A; Athenstaedt and Daum, 1997; Athenstaedt et al., 1999; Benghezal et al., 2007; Jain et al., 2007). Further conversion of PA in the ER provides the precursor for most major phospholipids in the cell (Shen et al., 1996). These precursor coupled with inositol acts as the intermediate for PI synthesis and the phosphorylated derivatives PI4P, PI3P and their derivatives PI(4,5)P_2_ and PI(3,5)P_2_ (Paulus and Kennedy, 1960; Nikawa and Yamashita, 1984; Schu et al., 1993; Yoshida et al., 1994; Yamamoto et al., 1995; Shelton et al., 2003; Jin et al., 2014). In mammals (not yeast), PI(4,5)P_2_ can be converted to PI(1,4,5)P_3_ and DAG by PLC, which can then be used to synthesize more PA (Figure 2B; Rhee, 2001).

The lipid precursors that produce phosphoinositides also yield PS, which is then converted to PE and eventually PC (Letts et al., 1983; Kiyono et al., 1987; Kodaki and Yamashita, 1987; McGraw and Henry, 1989; Clancey et al., 1993; Trotter et al., 1995). When ethanolamine and choline are exogenously added, PE and PC can also be produced via the Kennedy pathway, which serves as an independent means of PC synthesis (Nikawa et al., 1986; Hjelmstad and Bell, 1987, 1991; Tsukagoshi et al., 1987; Hosaka et al., 1989; Min-Seok et al., 1996; Patton-Vogt et al., 1997; Henry and Patton-Vogt, 1998; McMaster, 2018). In yeast, the PLD homolog Spo14p can hydrolyze PC to its choline and PA subunits and then the Kennedy pathway can reuse those precursors to regenerate PC (Patton-Vogt et al., 1997; Xie et al., 1998; Kim et al., 1999). ER-PM MCSs appear to coordinate at least part of the interdependencies between these biosynthesis pathways (Figure 2A; Omnus et al., 2016, Quon et al., 2018).

In Drosophila photoreceptors, the lipid transfer protein “RdgB”α maintains the level of PI(4,5)P_2_ in the PM. RdgBα transfers PA from the PM to ER and then reciprocally transfers ER-synthesized PI back to the PM where it serves as a PI(4,5)P_2_ precursor (Yadav et al., 2015; Cockcroft and Raghu, 2016; Cockcroft et al., 2016). This intermembrane transfer maintains signaling transduction upon photoreception, when high PLC activity causes rapid PI(4,5)P_2_ turnover. Phospholipid exchange occurs between ER and the PM at RdgBα-enriched ER-PM MCSs (Cockcroft and Raghu, 2016; Cockcroft et al., 2016). The clustering of RdgBα at these ER-PM contact sites involves its FFAT motif, which enables RdgBα recruitment by Drosophila VAP-A (Yadav et al., 2018). Moreover, the absence of VAP-A leads to RdgBα mis-localization, preventing it from performing its function at ER-PM MCSs thereby disrupting phototransduction (Yadav et al., 2018).

In mammalian cells, the response to PA production involves the translocation of the lipid transfer protein Nir2, the human RdgBα ortholog, from the Golgi to the PM (Kim et al., 2013). At ER-PM MCSs, Nir2 associates with VAP-B. Nir2 transfers PI from the ER to the PM while reciprocally transferring PA in the PM back to the ER. The PA transported to the ER contributes to the generation of more PI and other phospholipids in the ER. This lipid exchange also generates a PA gradient that allows PLC and PLD in the PM to generate more PA from PI(4,5)P_2_ and PC, respectively (Kim et al., 2013; Chang and Liou, 2015; Chen et al., 2019). In turn, PI transported to the PM by Nir2 can now generate PI4P and PI(4,5)P_2_ in the PM. When in excess, DAG generated from PI(4,5)P_2_ can also be transported from the PM to the ER via E-Syt tether proteins or by vesicular transport (Igal et al., 2001; Saheki et al., 2016). The control of PI(4,5)P_2_ signaling is in part coupled to Ca^2+^ regulation through the integrated functions of Nir2, STIM1, and E-Syt1 at ER-PM MCSs. Membrane contact is induced by E-Syt1 upon increases in cytosolic Ca^2+^, whereas STIM1 translocation to ER-PM MCSs is induced by ER Ca^2+^ depletion, which both stimulate Nir2 PA production in the PM (Chang and Liou, 2016). A similar PA/PI(4,5)P_2_ regulatory cycle responds to glucose levels in other cell types. In pancreatic β-cells when glucose levels are elevated, TMEM24 plays a similar role to Nir2 (Lees et al., 2017). TMEM24 transfers PI from the ER to the PM which can be converted to PI(4,5)P_2_ in the PM (Lees et al., 2017).

In mammalian cells, ORP5 and ORP8 act as PS/PI4P exchangers at ER-PM MCSs to maintain phospholipid pools across these membranes (Chung et al., 2015; Ghai et al., 2017). ORP5/8 binds to the ER-localized VAP proteins, where they transfer PS from the ER to the PM and PI4P in the opposite direction (Chung et al., 2015). Within the ER, the lipid phosphatase Sac1 dephosphorylates PI4P and the generated PI is again transferred by Nir2 to the PM in exchange for PA (Nemoto et al., 2000; Liu et al., 2009). Mammalian E-Syt2 recruits Sac1 to ER-PM MCSs for this activity (Dickson et al., 2016). ORP5/8-dependent PS/PI4P exchange is regulated by low levels of PM PI4P and PI(4,5)P_2_ causing ORP5/8 detachment from the PM to cease PI4P transport to the ER (Sohn et al., 2018). When PI(4,5)P_2_ levels are elevated in the PM, ORP5 transports excess PI4P out of the PM to normalize and maintain PM PI4P homeostasis (Figure 2B; Chung et al., 2015; Ghai et al., 2017; Sohn et al., 2018).

### Yeast ER-PM Contact Sites Represent a Nexus for Phospholipid Regulation

In yeast cells, ER-PM MCSs are also major determinants of phospholipid regulation. Akin to ORP5/8 in mammalian cells, yeast Osh6p/7p is recruited to ER-PM MCSs by the tether Ist2p, where Osh6p transfers PS in the ER to the PM for the reciprocal exchange of PM PI4P back to the ER (Maeda et al., 2013; Moser von Filseck et al., 2015a; D’Ambrosio et al., 2020). PE transported to the PM is converted to PC by the ER-localized Opi3p (Kodaki and Yamashita, 1987; McGraw and Henry, 1989). Another Osh protein, Osh3p, facilitates Opi3p recruitment to ER-PM MCSs where Opi3p acts *in trans* by reaching from the ER to generate PC in the PM (Figure 2A; Tavassoli et al., 2013; Pawlik et al., 2020). In Δ-s-tether cells lacking ER-PM MCSs, Opi3p cannot synthesize PC *in trans* and PC synthesis is disrupted (Quon et al., 2018). Supplying Δ-s-tether cells with supplemented choline improves cell growth likely by producing PC via the alternate Kennedy pathway (Quon et al., 2018).

In the PM, the PLD homolog Spo14p converts PC to PA but unlike mammalian cells, yeast do not have an established Nir2 homolog that transports PA back to the ER (Sreenivas et al., 1998; Xie et al., 1998). Instead, in the PM the lipid phosphatase App1p converts excess PA to DAG. In the ER, the PA phosphatase Pah1p uses PA as a substrate to produce DAG, which can be utilized for PE and PC synthesis by the Kennedy pathway (Figure 2A; Ganesan et al., 2015). At the same time, the lipid kinase Stt4p converts PI in the PM to PI4P, which is thereafter changed to PI(4,5)P_2_ by the PI5 kinases, Mss4p (Carman and Han, 2011).

For its role in PI4P regulation, it was suggested that the ER-localized Sac1p phosphatase acts *in trans* to eliminate PI4P in the PM (Stefan et al., 2011). The deletion of *SAC1* and the elimination of ER-PM tethers both aberrantly accumulate PI4P in the PM (Stefan et al., 2011; Quon et al., 2018). One possibility is that Osh3p and Scs2p recruit and activate Sac1p at MCSs in order to reach out to the PM from the ER (Stefan et al., 2011). In conjunction with ORP5-mediated transfer PI4P from the PM, an alternative model proposes that Sac1 in mammalian cells turns over PM PI4P delivered to the ER in *cis* (Zewe et al., 2018). In the same study, Sac1 was able to act *in trans* when fused with an additional 6−7.5 nm linker peptide between the catalytic domain and transmembrane domain of Sac1p, which the enabled Sac1 to bridge the distance between the ER to the PM to turn over PI4P (Zewe et al., 2018). Consistent with these findings, the deletion of *SAC1* in Δ-s-tether yeast cells results in synthetic lethality, which suggests that *SAC1* and ER-PM tethers act in parallel but functionally independent pathways (Quon et al., 2018). Moreover, when ER-PM contact is reintroduced in Δ-s-tether cells using an artificial ER-PM staple, the PM is still enriched in PI4P and does not re-establish the normal cellular distribution of PI4P, which is absent in the PM of mother cells (Quon et al., 2018). This would argue that forcing membrane contact between the ER and PM is not sufficient for Sac1p to act *in trans*, at least not effectively.

As with mammalian ORPs, the seven yeast ORPs Osh1p-Osh7p together also affect PI4P levels (Stefan et al., 2011). The Osh family of proteins share at least one overlapping essential function and eliminating all Osh proteins increases cellular PI4P by ∼20-fold over wild-type cells (Beh and Rine, 2004; Stefan et al., 2011). This accumulation is even greater than in *sac1*Δ cells where PI4P levels only increase ∼6 fold (Rivas et al., 1999). Give that more PI4P accumulates in Osh-depleted cells, the Osh protein family affects PI4P regulation beyond just Sac1p-dependent PI4P dephosphorylation. Osh proteins might also regulate other PI4P phosphatases or facilitate PI4P consumption and conversion into greater phosphorylated phosphoinositide species.

In Δ-s-tether cells, the lack of ER-PM MCSs leads to increased DAG levels, decreased levels of PA and PA-derived phospholipids, and decreased amounts of sphingolipids (Quon et al., 2018). This result suggests that DAG generation of PA is inhibited, and some aspect of PA biosynthesis is dependent on ER-PM MCSs. Because DAG is readily consumed in the synthesis of phospholipids and TAG, DAG generally represents a minor fraction of total membrane lipids (Ejsing et al., 2019). In growing yeast cells, however, DAG is enriched in the vacuolar membrane and a minor DAG pool resides in the ER membrane (Ganesan et al., 2019). The pool size and trafficking of DAG is also affected by other lipids including PS and also sphingolipid synthesis, which generates DAG (Figure 2A; Ganesan et al., 2019). In yeast strains defective in sphingolipid synthesis, DAG is primarily localized to the PM rather than the vacuolar membrane (Ganesan et al., 2019). As such, ER-PM MCSs might affect DAG pools by modulating PS trafficking or sphingolipid synthesis, and/or by repressing the normal regulation of DAG-PA interconversion. In general, this regulatory control over phospholipid synthesis and distribution impacts membrane structure and functional activities.

### ER-PM Contact Sites as Zones for Sterol Biosynthesis and Membrane Exchange

The original finding that PAM fractions are enriched in ER sterol biosynthetic enzymes, also suggests an intimate relationship between ER sterol synthesis and those areas of the ER associated with the PM (Pichler et al., 2001). Moreover, the inactivation of sterol synthesis in yeast induces ER-PM MCSs, resulting in the nearly complete coverage of the PM with cER (Quon et al., 2018). In addition to potential sterol exchange at ER-PM MCSs, additional mechanisms clearly contribute to sterol transport.

Through exocytosis, some sterol is sorted and transferred to the PM by vesicular transport (Surma et al., 2011). In the absence of vesicular transport, however, sterols are still delivered to the PM from their site of production in the ER (Kaplan and Simoni, 1985). A conserved aspect of this “non-vesicular” transfer mechanism is that it involves equilibrative exchange between the ER and PM (Kaplan and Simoni, 1985; Menon, 2018). Even though sterol transport is non-directional, sterols ultimately concentrate within the PM because its lipid composition is preferable for sterol inclusion (Baumann et al., 2005; Menon, 2018). As presumptive sites for ER-PM sterol exchange, ER-PM MCSs might reduce the intermembrane gap necessary for membrane-bound lipid transfer proteins to transport sterols. In yeast, the necessity of ER-PM MCSs for sterol exchange has been directly tested and, despite a defect in sterol internalization, mutant cells lacking the seven primary tethers still retain normal equilibrative sterol exchange between the two membranes (Quon et al., 2018).

Plasma membrane fractions from either wild-type or Δ-s-tether cells indicate that the subcellular distribution or ergosterol (the yeast equivalent of cholesterol) is unaffected by the absence of ER-PM contact sites (Quon et al., 2018). However, retrograde transport of sterols from the PM back to the ER is moderately defective in Δ-s-tether cells (Quon et al., 2018). Using transport-coupled esterification of exogenous sterols as an assay, retrograde transport is slowed ∼4-fold as the imported sterol moves from the PM to the ER and then lipid droplets, following esterification. Measuring sterol transport in the opposite direction, a pulse-chase analysis of *de novo* synthesized [^3^H]ergosterol indicated that the half time of sterol transfer from the ER to the PM is ∼10 min in both wild-type and Δ-s-tether yeast (Quon et al., 2018). These results suggest a limited role of ER-PM MCSs in equilibrative sterol exchange.

ER-PM MCSs and membrane-bound sterol transfer proteins might facilitate sterol exchange if they act in concert with other independent transport mechanisms. For instance, an overlapping mechanism for sterol delivery to the PM is vesicular transport. However, blocking exocytosis in △-s-tether cells has no significant effect on the transfer of sterols from the ER to PM (Quon et al., 2018). As another redundant exchange pathway, soluble sterol transfer proteins might act as a compensatory mechanism in the absence of sterol exchange at ER-PM MCSs.

In yeast cells, Osh4p is the most abundant yeast ORP that binds both sterols and PI4P (Im et al., 2005; de Saint-Jean et al., 2011). In liposome transfer assays, Osh4p was shown *in vitro* to exchange sterols and PI4P (de Saint-Jean et al., 2011; Moser von Filseck et al., 2015b). However, *in vivo* sterol transfer assays have not confirmed this activity in living cells, at least between the ER and PM (Georgiev et al., 2011; Quon et al., 2018). Osh4p associates with the Golgi and post-Golgi exocytic vesicles and its transfer activities are likely more pertinent in that cellular context (Fairn et al., 2007; Alfaro et al., 2011). In fact, the entire family of Osh proteins is dispensable for ER to PM sterol transfer, though specific Osh proteins affect phosphoinositide metabolism at ER-PM MCSs (Georgiev et al., 2011; Stefan et al., 2011). The role of Osh4p in phosphoinositide regulation, as opposed to sterol transfer, is the probable basis for the lethality of deleting *OSH4* in Δ-s-tether cells (Quon et al., 2018). In sterol transfer assays using a conditional *osh4* Δ-s-tether mutant, no compounding defect in ER-PM sterol exchange was detected (Quon et al., 2018). The fact that the absence of ER-PM contact sites has no impact on ER to PM sterol transfer is not due to compensatory transport by vesicular transport or Osh4p, which is the predominant sterol-binding protein in yeast. Although the equilibrative mechanism of sterol exchange within yeast is unclear, the import of exogeneous sterols is affected by ER-PM MCSs and associated sterol transfer proteins.

The StARkin family possess StART domains, or StART-like domains, which mediate lipid and sterol transfer (Gatta et al., 2018; Horenkamp et al., 2018; Jentsch et al., 2018; Tong et al., 2018). In budding yeast, there are six StART domain homologs that include Lam1p-4p, which are ER membrane proteins that can be found at various organelle contact sites (Gatta et al., 2015). In Lam proteins, within the StART-like domain, a hydrophobic cavity can accommodate a sterol ligand (Tong et al., 2018). Yeast cells lacking Lam2p (also known as Ysp2p) exhibit reduced esterification rates of imported sterol, indicating a role in exogenous sterol uptake (Roelants et al., 2018; Tong et al., 2018). Lam2p localizes to spots both in cytoplasmic ER and where cER and PM are associated, though distinct from where other functional tethers are observed (Gatta et al., 2015; Quon et al., 2018). In fission yeast, the Lam homolog Ltc1p is also localized to sites on perinuclear ER and the cell cortex, where it is involved in retrograde sterol transfer (Marek et al., 2020). At the cell cortex, Ltc1p facilitates an actin-dependent sterol transfer either directly from the PM to endosomes, or indirectly through the ER as an intermediate (Marek et al., 2020).

The mammalian homologs of the Lam/Ltc protein family are GRAMD1a, b, and c, GRAMD2a and b (also known as GRAM3). GRAMD1a, b, and c contain both StART-like sterol transfer domains and PH-like GRAM (Glucosyltransferases, Rab-like GTPase activators and Myotubularins) sterol-sensing domains, while GRAMD2a and b contain only GRAM domains (Horenkamp et al., 2018; Sandhu et al., 2018; Naito et al., 2019). At ER-PM contacts, GRAMD2a co-localizes with the E-Syt2 and E-Syt3 tethers (Besprozvannaya et al., 2018). In response to PM cholesterol accumulation, the N-terminal GRAM domain of GRAMD1b (also known as Aster-B protein) binds PS and cholesterol, which recruits GRAMD1b to ER-PM MCSs where it mediates retrograde cholesterol transport (Sandhu et al., 2018; Naito et al., 2019). PM cholesterol accumulation cannot be cleared when GRAMD1 proteins are eliminated, indicating that they facilitate cholesterol transport out of the PM and into the cell (Naito et al., 2019). As sites of lipid transfer or regulation, ER-PM MCSs affect the distribution and metabolism of both sterols and phospholipids, which directly impacts PM and ER function and morphology.

## ER-PM Contact Sites Confer Unique Membrane Structures for cER Extension and Mitotic ER Inheritance

In neurons, continuous smooth “thin” ER tubules extend into axons. At synaptic boutons, the tubules branch and wrap around several organelles and synaptic vesicles at nerve termini (Wu et al., 2017). ER-PM MCS tether proteins have additional functions in controlling axonal ER morphology and regulating neurite outgrowth. During growth cone navigation, STIM1 mediates an interaction between ER and microtubules through direct interactions with EB proteins attached to the plus-end of microtubules (Grigoriev et al., 2008; Pavez et al., 2019). This interaction recruits microtubules to the motile growth cone periphery, which remodels ER for axon guidance and localized Ca^2+^ signaling (de Juan-Sanz et al., 2017; Pavez et al., 2019).

In plants, the major conserved families of ER-PM tethers affect plant-specific structures. Plant cells are linked together through distinctive ER-PM MCS structures at plasmodesmata, where the compressed ER of the desmotubules extend from one cell into the next (Grison et al., 2015). Both SYT1 and VAP27 are the implicated tethers that connect the plasmodesmata to the closed apposed PM, which also mediates stress tolerance and plant defenses (Levy et al., 2015; Wang et al., 2016).

SYT1 and VAP27 are also involved in anchoring the PM to the cell wall during plasmolysis, in which plant cells exposed to hyperosmotic stress lose water causing the cell membrane release from the cell wall (Schapire et al., 2008; Wang et al., 2016). This localization to stretched regions of the PM at the tips of Hechtian strands suggests that VAP27 and SYT1 ER-PM MCSs provide additional mechanical support to the PM. How these tethers interact with the cell wall is not well understood (Schapire et al., 2008; Wang et al., 2016). These findings show how ER-PM MCSs generally regulate the dynamics of membrane structure and distribution.

In both yeast and higher eukaryotes, the ER is an essential organelle that is not synthesized *de novo*. In a number of cell types, cER redistribution along the PM is cell-cycle regulated. For example, in mammals, the ER is redistributed into cortical clusters at the cortex during oocyte maturation, which facilitates Ca^2+^ storage and regulation (FitzHarris et al., 2003; Kim et al., 2014). In *S. cerevisiae*, cER segregation from mother cells to growing daughter cells is tightly regulated event during the cell cycle (Fehrenbacher et al., 2002; Du et al., 2004). At the bud tip of the daughter cell, once cER is established by its attachment to the PM, cER expands through the entire bud to form a complex ER network (Du et al., 2006). cER attachment to the PM is therefore an inherent necessity for ER inheritance, which is essential for proper cell division.

In growing yeast, Ice2p facilitates the attachment and segregation of cER into the bud; *ICE2* deletion causes defects and abnormalities in cER distribution (Estrada de Martin et al., 2005). Although Ist2p and the Tricalbins Tcb2p and Tcb3p are also localized to the cER in budding daughter cells, it is because their mRNAs are specifically targeted into the bud (Shepard et al., 2003). The transcripts for these tethers are then translated within the bud presumably for the expansion of cER already inherited from the mother cell via Ice2p. In contrast, Scs2p is ubiquitously present in all forms of ER, which provides multiple paths for its inheritance into the daughter cell. In fact, the combined deletions of *SCS2* and *ICE2* negatively impacts cER accumulation in the bud more than mother cells (Tavassoli et al., 2013). These results indicate that Scs2p and Ice2p are tether proteins that are more involved in facilitating cER inheritance, whereas Ist2p and Tricalbins are more relevant to generalized cER expansion.

## ER-MCSs Promote ER Membrane Stress Responses

Delays in cER inheritance induce ER stresses that elicit a complex cell cycle response involving the Slt2p MAP kinase (Babour et al., 2010). This “ER surveillance pathway” provides monitoring system for the functional capacity and segregation of ER prior to cell division. The sensor for Slt2p activation in this pathway is Slg1/Wsc1p, a PM transmembrane domain protein that is otherwise involved in the maintenance of cell wall integrity (Verna et al., 1997; Philip and Levin, 2001). Through Slg1/Wsc1p and Slt2p, ER stress ultimately delays the transfer of cER into daughter cells to maintain mother cell viability until ER functionality can be restored for both mother and daughter bud. In the event of ER stress, the domain restriction of cER also prevents the spread of unfolded proteins to daughter cells and isolates the contagion of ER stress to the mother cell (Babour et al., 2010).

As one of the hallmark events in “ER surveillance,” septin filaments that form a ring around the bud neck are stabilized during ER stress and do not disperse as required for cytokinesis (Babour et al., 2010). Normally, septins at the PM around the bud neck form a structure that separates the mother cell from the growing daughter bud, but it also restricts cER between these two domains (Babour et al., 2010; Chao et al., 2014; Clay et al., 2014). By preventing the dispersal of the septin ring during ER stress, the ER surveillance pathway stops “damaged ER” from entering the daughter cell, which also delays cytokinesis. The physical interactions between the septin Shs1p, the ER-PM tether protein Scs2p, and the polarisome-binding protein Epo1p enforce the diffusional barrier to cER transfer (Chao et al., 2014). This complex of proteins represents another bona fide multi-subunit ER-PM tether that is ultimately required for controlling ER polarization into daughter buds.

Although the ER surveillance pathway is affected by ER stress, it is distinct and independent from the well-established “UPR” pathway (Babour et al., 2010). Whereas the UPR pathway is *IRE1*-dependent and is induced by the ER-luminal accumulation of unfolded proteins, the ER surveillance pathway is triggered by a transient increase in sphingolipids and ceramides, which in turn a transient phosphorylation and activation of Slt2p (Piña et al., 2018). It is unclear how sphingolipid biosynthesis is induced by ER stress, though it seems to involve the SPOTS (Serine Palmitoyltransferase, Orm1/2, Tsc3, and Sac1) complex, and more specifically Orm1p and Orm2p, which negatively regulate sphingolipid biosynthesis (Breslow et al., 2010; Han et al., 2010). How the ER surveillance pathway is affected by wider issues of ER-PM tethering is also unclear. Scs2p and Ice2p are directly involved in ER inheritance, and cells that lack ER-PM MCSs exhibit increases in ceramide levels though sphingolipid levels are substantially decreased (Quon et al., 2018). How these particular changes in ceramide and sphingolipid synthesis impact ER surveillance is unknown.

In yeast, major disruptions in ER-PM contact leads to UPR activation via Ire1p, the ER membrane-bound sensor (Manford et al., 2010). Ire1p activation of the Hac1p transcription factor induces genes for protein folding, protein degradation, anti-oxidative stress and lipid/inositol metabolism (Travers et al., 2000; Kimata et al., 2005). Driven by lipid biosynthesis, UPR activation also increases ER membrane expansion in the cytoplasm, though the ER at the cortex is unaffected (Schuck et al., 2009). This UPR-driven ER expansion requires the Ino2p/4p transcription factors that are positive regulators of phospholipid biosynthesis. Expansion and increased volume of the ER might facilitate protein folding, and in this context ER-PM MCSs might affect lipid biosynthesis that is in turn necessary for ER expansion (Apetri and Horwich, 2008; Schuck et al., 2009). However, a recent study showed that the canonical UPR pathway is independently induced by lipid bilayer stress and proteotoxic stress (Ho et al., 2020). Changes in the ER lipid composition affecting the PC/PE ratio induce a strong UPR bilayer stress that is attenuated by choline, but unaffected by the protein aggregation inhibitor 4-phenylbutyric acid. In either the yeast or mammalian homolog, removal of the Ire1p ER luminal domain, which mediates the response to ER proteotoxic stress, does not affect UPR activation in response to cellular perturbations disrupting lipid regulation (Volmer et al., 2013; Ho et al., 2020). Instead, the Ire1p transmembrane domain induces a specific UPR program in response to lipid bilayer stress that induces genes to restore lipid and ER membrane homeostasis (Figure 2A; Ho et al., 2020). Removal of yeast ER-PM MCSs causes significant lipid homeostasis defects as well as UPR activation (Manford et al., 2012; Quon et al., 2018). It seems probable that the phospholipid defects observed in yeast ER-PM tether mutants induce this independent lipid bilayer-stress UPR program.

In mammalian cells, the homologous IRE1-dependent pathway activates the UPR-dependent transcription factor XBP1, which also induces ER expansion and phospholipid biosynthesis (Sriburi et al., 2004). Through XBP1, UPR activation enhances PC synthesis by increasing CCT and CPT biosynthetic activities, in an indirect post-transcriptional mechanism (Figure 2B; Sriburi et al., 2004). ATF6 represents another UPR sensor, that mediates increased PC synthesis and ER expansion. Although ATF6 signaling converges on XBP1, increases in phospholipid synthesis by ATF6 are partially XBP1 independent (Bommiasamy et al., 2009). The PERK is yet another major mammalian ER sensor for UPR activation during ER stress (Schröder and Kaufman, 2005). Both Ire1 and PERK transmembrane domains act as UPR signaling sensors in response to membrane perturbation (Volmer et al., 2013). In a UPR-independent mechanism, however, PERK also affects Ca^2+^-induced ER stress and regulates ER-PM MCSs assembly through the redistribution of STIM1 and E-Syt1 ER tethering proteins (van Vliet et al., 2017). Ca^2+^ depletion from the ER stores, and the subsequent elevation of cytosolic Ca^2+^, triggers PERK dimerization and through its interaction with the actin-binding protein Filamin A, causing a remodeling of the association between F-actin and the ER (van Vliet et al., 2017). This change in cortical actin-ER organization allows greater ER access to the PM, which in turn stabilizes STIM1 and E-Syt1 contact with the PM. PERK also exhibits a very direct role in lipid biosynthesis due to its intrinsic lipid kinase activity, which synthesizes PA upon UPR induction (Bobrovnikova-Marjon et al., 2012). In addition to the impact of PA as a signaling ligand, the PA synthesized serves as an anabolic substrate for other phospholipids (Bobrovnikova-Marjon et al., 2012; Foster, 2013).

### Phosphoinositide Signaling and PM Membrane Stresses Affect Plant ER-PM Tethers

**FIGURE 3:**
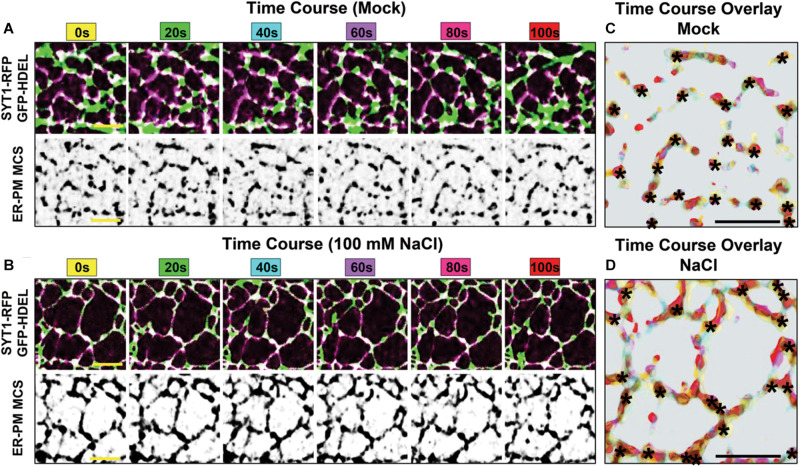
Environmental regulation and dynamics of SYT1-labeled ER-PM MCS in Arabidopsis seedlings. **(A,B)** Time course co-localization of SYT1-RFP ER-PM MCSs (purple) with the luminal ER marker GFP-HDEL (green). 5-day-old Arabidopsis cotyledon epidermal cells where treated with mock **(A)** or ionic stress (100 mM NaCl) **(B)** for 8 h prior to imaging. For the subsequent 100 s time course overlay, SYT1-RFP is shown corresponding black and white panels. **(C,D)** The time course overlay of SYT1-RFP dynamics in A and B is shown in panels **(C,D)**, respectively. Each color in the overlay represents the position of the SYT1-RFP signal at the corresponding 20 s interval. Asterisks mark where SYT1-RFP nodes are static throughout the time course. Bars = 5 mm.

In Arabidopsis, knock-out mutations in SYT1 render cells hypersensitive to environmental conditions that trigger membrane stress such as mechanical, cold, and ionic damage (Schapire et al., 2008, 2009; Yamazaki et al., 2008; Pérez-Sancho et al., 2015, 2016). These phenotypes suggest that ER-PM MCSs function is required for structural support further buttressed by the ER and the cytoskeleton. This hypothesis is further supported by dynamic phosphoinositide-mediated changes in ER-PM MCSs observed in Arabidopsis in response to ionic stress and rare earth elements (Lee et al., 2019, 2020; Figure 3).

At the molecular level, the ionic stress response induces PI(4,5)P_2_ accumulation, which promotes the electrostatic binding of the C-terminal C2 domains of ER-anchored SYT1 to the PM (Pérez-Sancho et al., 2015). This slow, but highly dynamic process effectively increases ER-PM association, which promotes a protective response that enables the PM to withstand ionic stress. Changes in phosphoinositide composition and ER-PM MCS assembly are specifically induced by stresses that inhibit PM Ca^2+^ channels. Perturbations of Ca^2+^ channel activity by rare earth elements trigger the PM accumulation of PI4P and results in a three- to four-fold increase in SYT1/SYT5 ER-PM MCSs (Lee et al., 2020). This mechanism demonstrates that the coordination of Ca^2+^ and phosphoinositide signals, together with ER-PM MCSs, sustains plant growth under suboptimal stress conditions (Bayer et al., 2017).

The stress-induced changes in phosphoinositides also highlight a role for ER-PM MCS as molecular platforms for other phosphoinositide-dependent processes. As first shown in mammalian cells, E-Syts ER-PM at MCSs become sites for PI3P synthesis where they promote autophagosome biogenesis (Nascimbeni et al., 2017). In plants, the presence of an autophagy-dependent pathway starting at ER-PM MCSs involves the interaction among the Arabidopsis AtEH/Pan1 (homologs of the yeast ARP2/3 complex activator, Pan1p), the actin cytoskeleton, and the ER-PM MCS resident proteins VAP27-1 and VAP2-3 (Stefano et al., 2018; Wang and Hussey, 2019; Wang et al., 2019). These studies feature an important role for plant and animal ER-PM MCS in the targeted removal and recycling of endocytic machinery as a cellular adaptive mechanism to nutrient deprivation.

## Conserved ER-PM Tethers are Implicated in Disease Etiology

The prevalence of ER-PM association in neuron cell bodies might explain the etiological link between ER-PM MCS dysfunction and neurodegenerative disorders such as HSPs, SMA, and ALS (Nishimura et al., 2004; Blackstone, 2012, 2018; Darbyson and Ngsee, 2016). HSPs are a group of genetically heterogeneous neurodegenerative diseases that disrupt the long axons of the corticospinal pathway that affect motor function (Blackstone, 2018). Many HSP mutations disrupt genes encoding proteins that organize and maintain neuronal ER structure (Boutry et al., 2019; Fowler et al., 2019). These proteins include reticulons, atlastin and other ER-shaping factors, though a specific ER-PM tether has yet to be implicated in an HSP disorder (Fowler et al., 2019). The inheritable disorders ALS8 and late-onset SMA are caused by missense mutations in VAP-B (Nishimura et al., 2004). VAP-B is involved in ER-PM tethering but because VAP-B also resides at several different organelle contact sites, it is not clear whether ER-PM tethering is necessarily or exclusively involved in ALS8 pathology. Secretory and phosphoinositide defects in VAP-B/ALS8 mutants can be rescued by overexpression of OSBP (Oxysterol-binding protein) in Drosophila, or ORP3 (OSBP-related protein 3) in HeLa cells (Moustaqim-Barrette et al., 2014; Darbyson and Ngsee, 2016). Indeed, the ORP family of proteins are themselves implicated in the regulation of multiple organelle MCSs within cells (Pietrangelo and Ridgway, 2018). Phosphoinositide regulation is a recurrent theme in VAP and ORP activities at MCSs.

The Anoctamin/TMEM16/Ist2p ion channel/lipid scramblase family is also directly involved in a wide variety of diseases (Whitlock and Hartzell, 2017). In particular, defects in ER-localized ANO5 (Anoctamin 5/TMEM16E) transmembrane protein cause adult-onset muscular dystrophy (Bolduc et al., 2010). In MMD3 patient-derived myoblasts, mutant ANO5 failed to repair PM injury due to the compromised ability of the ER to remove Ca^2+^ at PM damage (Chandra et al., 2019). Given that ER-PM tethering is essential for cellular Ca^2+^ homeostasis in mammalian cells, these results suggest a role for ANO5 at regions of ER-PM association.

The cell-cell movement of viruses in plants provides another very different example of how ER-PM MCSs affect disease. Plant homologs of the E-Syt family are recruited to facilitate infection of several diverse viruses, including Tobamovirus (Levy et al., 2015). At these sites, the cER is remodeled to form viral replication sites on the PM adjacent to plasmodesmata, which form syncytial connections between cells that enables viral spread. *A. thaliana* infection by powdery mildew fungi is also severely inhibited in *syt1* mutant plants, but in this case the role of the plant E-Syt homolog SYT1 seems to involve endocytosis (Kim et al., 2016; Yun et al., 2016). In the absence of SYT1, there is an enrichment of PM pools of *PEN1* (*PENETRATION1*), which otherwise cycles between endosomes and the PM and regulates a distinct immune secretory pathway (Kwon et al., 2008; Lewis and Lazarowitz, 2010; Reichardt et al., 2011; Kim et al., 2016).

In mammalian cells, E-Syt1 and E-Syt3 interacts with the HSV-1 glycoprotein gM (El Kasmi et al., 2017). In this case, viral release and cell-to-cell spreading through virus-induced syncytia is actually repressed by these E-Syts. How other mammalian E-Syts regulate or modulate HSV-1-induced fusion events requires further analysis. Across species, however, depending on cellular context, ER-PM tether proteins present a multitude of mechanisms in disease pathologies.

## Unresolved Paradoxes

Despite their importance to membrane and lipid regulation, or perhaps because of it, ER-PM tethers are not essential for growth; these proteins are functionally redundant with other cellular pathways. In yeast, the elimination of nearly all cER-PM association is not lethal (Quon et al., 2018). The resulting membrane dysfunction in these cells is presumably ameliorated by compensatory changes in lipid synthesis and/or membrane stress responses. In mice, the elimination of all E-Syts has no apparent effect on basic cellular functions (Sclip et al., 2016; Tremblay and Moss, 2016). However, mice lacking all E-Syts show a compensatory increase in the expression of Orp5/8, Orai1, STIM1 and TMEM110, which might adaptively correct any tethering deficiency (Tremblay and Moss, 2016). Mouse embryonic fibroblasts in which E-Syt2 and E-Syt3 are eliminated are more sensitive to stringent culture conditions and oxidative stress (Herdman et al., 2014). Dynamic changes in ER-PM MCS density also appears to ameliorate stresses on the ER and PM. Perhaps the essential importance of ER-PM tether proteins is best revealed under conditions of membrane stress.

Phosphoinositide regulation and signaling is a recurrent theme whenever ER-PM MCS function is investigated. Whether induced by stress or as a necessary part of phosphoinositide cycling between membranes, ER-PM MCSs, with ancillary regulators and effectors like Sac1p and the ORP proteins, are major regulators of PI4P, PI(4,5)P_2_, and even PI3P (Stefan et al., 2011; Stefan, 2020). In turn, ER-PM MCSs serve as important hubs for many phosphoinositide-dependent processes relating to PM dynamics, growth, and membrane repair. Together with the general regulation of phospholipid biosynthesis, these processes appear to be coordinated with the regulation of other cellular structures such as the cytoskeleton. The cytoskeleton often represents a barrier to organelle membrane interactions and is an organizing structure for controlling ER-PM MCS dynamics. Indeed, investigations into the wider role of ER-PM MCSs in controlling cellular structure during the cell cycle might supplant studies of their role in phospholipid and membrane regulation.

## Author Contributions

MZ, AN, ARa, ARo, and CB wrote the manuscript. ARo and CB prepared the figures. All authors contributed to the article and approved the submitted version.

## Conflict of Interest

The authors declare that the research was conducted in the absence of any commercial or financial relationships that could be construed as a potential conflict of interest.

## Footnotes

1 Using the cytoplasmic volume determined for a prolate unbudded yeast cell (excluding volumes of major organelles), and the volume occupied by the ER-association with the PM (40% cortical coverage with a 35 nm thickness, corresponding to the average ER-PM gap distance), the ratio of cER:cytoplasm volume is ∼0.02. In other words, 2% of untethered cER would be predicted to still be in the PM vicinity (if evenly dispersed throughout the cytoplasm). These calculations are qualified by the assumption that untethered ER is unstructured and not elastic, which is generally consistent with experimental observations.

## Abbreviations

Δ -s-tether: Δ -super-tether
ALS: amyotrophic lateral sclerosis
cER: cortical endoplasmic reticulum
CCT: choline cytidylyltransferase
CPT: cholinephosphotransferase
DAG: diacylglycerol
ER: endoplasmic reticulum
ERMES: ER-mitochondria encounter structure
E-Syt: extended synaptotagmin
FFAT: two phenylalanines in an acidic tract
GRAM domain: Glucosyltransferases, Rab-like GTPase activators and Myotubularins domain
HOG: high osmolarity glycerol
HSPs: hereditary spastic paraplegias
MSMs: medium spiny neurons
LD: lipid droplets
MCSs: membrane contact sites
MSP domain: major sperm protein domain
OSBP: oxysterol-binding protein
ORP: OSBP-related protein
Osh: OSBP homolog
NVJ: nuclear-vacuolar junction
PA: phosphatidic acid
PAM: PM-associated membrane
PC: phosphatidylcholine
PE: phosphatidylethanolamine
PERK: PRK-like endoplasmic reticulum kinase
PH domain: pleckstrin homology domain
PI: phosphatidylinositol
PI4P: phosphatidylinositol-4-phosphate
PI(4,5)P_2_: phosphatidylinositol-4,5-bisphosphate
PM: plasma membrane
PS: phosphatidylserine
SERCA: sarco/endoplasmic reticulum Ca^2+^-ATPase
SMA: spinal muscular atrophy
SMP domain: synaptotagmin-like mitochondrial protein domain
SOCE: store-operated Ca^2+^ entry
SPOTS complex: serine palmitoyltransferase, Orm1/2, Tsc3, and Sac1 complex
StART domains: steroidogenic acute regulatory transfer domains
SYT: synaptotagmin
TAG: triacylglycerol
Tcb: tricalbin
TMEM protein: transmembrane protein
TULIP domain: tubular lipid binding domain
UPR: unfolded protein response
VAMP: vesicle-associated membrane protein
VAP: VAMP-associated protein
WT: wild type.
